# Stand Out in Class: restructuring the classroom environment to reduce sitting time – findings from a pilot cluster randomised controlled trial

**DOI:** 10.1186/s12966-020-00958-z

**Published:** 2020-04-29

**Authors:** Stacy A. Clemes, Daniel D. Bingham, Natalie Pearson, Yu-Ling Chen, Charlotte L. Edwardson, Rosemary R. C. McEachan, Keith Tolfrey, Lorraine Cale, Gerry Richardson, Mike Fray, James Altunkaya, Stephan Bandelow, Nishal Bhupendra Jaicim, Jo Salmon, David W. Dunstan, Sally E. Barber

**Affiliations:** 1 National Centre for Sport and Exercise Medicine, School of Sport, Exercise and Health Sciences, Loughborough University, Loughborough, UK; 2 NIHR Leicester Biomedical Research Centre, Leicester, UK; 3 Bradford Institute for Health Research, Bradford Teaching Hospitals Foundation Trust, Bradford, UK; 4 Diabetes Research Centre, University of Leicester, Leicester, UK; 5 Centre for Health Economics, University of York, Heslington, York, UK; 6 Loughborough Design School, Loughborough University, Loughborough, UK; 7 Department of Physiology, Neuroscience and Behavioral Sciences, St. George’s University, West Indies, Grenada; 8 Leicester Clinical Trials Unit, University of Leicester, Leicester, UK; 9 Institute for Physical Activity and Nutrition, School of Exercise and Nutrition Sciences, Deakin University, Melbourne, Australia; 10 Baker Heart and Diabetes Institute, Melbourne, Australia; 11 Mary MacKillop Institute for Health Research, Australian Catholic University, Melbourne, Australia

**Keywords:** Standing desks, Sit-stand desks, Primary/elementary school, Sedentary behaviour, Bradford, South Asian, Children, Health inequalities

## Abstract

**Background:**

Excessive sedentary behaviour (sitting) is a risk factor for poor health in children and adults. Incorporating sit-stand desks in the classroom environment has been highlighted as a potential strategy to reduce children’s sitting time. The primary aim of this study was to examine the feasibility of conducting a cluster randomised controlled trial (RCT) of a sit-stand desk intervention within primary school classrooms.

**Methods:**

We conducted a two-armed pilot cluster RCT involving 8 primary schools in Bradford, United Kingdom. Schools were randomised on a 1:1 basis to the intervention or usual practice control arm. All children (aged 9–10 years) in participating classes were eligible to take part. Six sit-stand desks replaced three standard desks (sitting 6 children) in the intervention classrooms for 4.5-months. Teachers were encouraged to use a rotation system to ensure all pupils were exposed to the sit-stand desks for > 1 h/day on average. Trial feasibility outcomes (assessed using quantitative and qualitative measures) included school and participant recruitment and attrition, intervention and outcome measure completion rates, acceptability, and preliminary effectiveness of the intervention for reducing sitting time. A weighted linear regression model compared changes in weekday sitting time (assessed using the activPAL accelerometer) between trial arms.

**Results:**

School and child recruitment rates were 33% (*n* = 8) and 75% (*n* = 176). At follow-up, retention rates were 100% for schools and 97% for children. Outcome measure completion rates ranged from 63 to 97%. A preliminary estimate of intervention effectiveness revealed a mean difference in change in sitting of − 30.6 min/day (95% CI: − 56.42 to − 4.84) in favour of the intervention group, after adjusting for baseline sitting and wear time. Qualitative measures revealed the intervention and evaluation procedures were acceptable to teachers and children, except for some problems with activPAL attachment.

**Conclusion:**

This study provides evidence of the acceptability and feasibility of a sit-stand desk intervention and evaluation methods. Preliminary evidence suggests the intervention showed potential in reducing children’s weekday sitting but some adaptations to the desk rotation system are needed to maximize exposure. Lessons learnt from this trial will inform the planning of a definitive trial.

**Trial registration:**

ISRCTN12915848 (registered: 09/11/16).

## Background

Advances in technology and changes to our environments have resulted in sedentary behaviour becoming ubiquitous within all settings of daily life. Sedentary behaviour is distinct from physical (in)activity and is defined as ‘any waking behaviour characterised by an energy expenditure ≤1.5 metabolic equivalents (METs) while in a sitting, reclining or lying posture’ [1]. In the UK, sitting is the most prevalent behaviour exhibited during waking hours in children, typically accounting for over 65% (~ 7.5 h/day) of waking time [2], with some children reportedly sitting for over 10 h/day [3]. Sedentary time is associated with an increased risk of a number of chronic conditions in adults, including cardiovascular disease, type 2 diabetes and all-cause mortality [4–7]. Whilst evidence of the associations of sedentary time with increased risk of adiposity/weight gain and clustered cardiometabolic risk in children is largely restricted to screen time [8], sedentary behaviours have been shown to increase across key transitions in children’s lives (e.g. from primary to secondary school) [9] and track into both adolescence [10] and adulthood [11]. Reducing children’s sitting time may therefore be important for the primary prevention of chronic diseases in adulthood [12].

The emergence of an increased cardiometabolic health risk profile in some population groups is evident during the first decade of life [13]. For example, British South Asian children have demonstrated higher glycated haemoglobin, fasting insulin and triglyceride and lower high-density lipoprotein-cholesterol levels compared to white British children as well as higher levels of fat mass percentage [14, 15]. Higher levels of sedentary behaviour (ranging between an additional 28 to 39 min/day) have also been observed in South Asian school-aged children (aged 6–11 years) in comparison to White British children [16, 17]. Given the links between sedentary behaviour and cardiometabolic risk [8], early interventions in such at-risk groups may help reduce health inequalities later in life.

The environments and social norms that children are exposed to have dominant influences on their activity behaviour [18]. Given children spend half of their waking hours at school, it is plausible that the school environment may be a critical influence on their health behaviour patterns [19–21] and be an appropriate setting for interventions [22], particularly in relatively deprived locations with higher levels of health inequalities. Indeed, there has been a growing interest in the use of sit-stand desks (desks which provide children with the opportunity to alternate their posture between sitting and standing) within the classroom environment as a tool to reduce sedentary behaviour. Classroom-based interventions have the potential to target health inequalities because they are accessible to all children [12].

Systematic and narrative reviews of sit-stand desk interventions within the classroom environment have concluded that this approach shows promise as an effective tool for reducing children’s sitting time and increasing movement. However the majority of studies included in these reviews have been feasibility trials or small-scale single-school pilot studies [23–25]. Knowledge of the impact of sit-stand desks on sedentary behaviour, markers of adiposity and pupil behaviour is currently limited by a lack of randomised controlled trials (RCTs) [26, 27], and relatively small samples (median sample size across studies: 45 [24, 26–30]). Furthermore, there has been a limited focus on the acceptability of this intervention approach in the form of qualitative feedback from teachers and pupils, and in understanding pupils’ experiences and responses (for example, in-class behaviour) to using sit-stand desks [26, 31, 32]. The above factors will be vital to establish prior to schools agreeing to the longer-term adoption of this strategy [23, 24, 26]. Limited research in this area has also been conducted within relatively deprived locations and/or higher-risk populations, such as South Asian children.

We have previously demonstrated the feasibility of incorporating sit-stand desks in the classroom environment over a 9-week period in a small non-randomised controlled study conducted within one UK primary school with children aged 9–10 years [33]. In this novel intervention, three standard desks (sitting six children) were replaced with six sit-stand desks in one classroom. The teacher (who received training in intervention delivery) rotated the children in the intervention classroom, using naturally occurring breaks between lessons to do so, to ensure each child was exposed to the desks for at least one hour/day. Children in a control group (within the same school) continued with their usual practice, and no environmental changes were made to their classroom. Reductions in total daily sitting time of 81 mins/day on weekdays (school days) after 9-weeks were seen in the intervention group. As part of this feasibility work, changes in sitting observed in the sample were compared to data from a related feasibility study conducted in a primary school in Melbourne, Australia [33]. Within the Melbourne-based study, every child in the intervention classroom had a sit-stand desk. No significant differences in reductions in weekday total sitting time were observed between studies, demonstrating the potential of this intervention, over the short-term, to reduce children’s daily sitting time irrespective of the different approaches to sit-stand desk provision employed.

This paper reports the findings of a pilot cluster RCT, conducted in a relatively socially deprived location within the UK. Rapid increases in sedentary time have been observed in children aged 11 years and above [34]. This study therefore targeted year 5 classrooms and involved children aged 9–10 years, with the goal of mitigating the typical rise in sedentary time seen during the transition into adolescence [9]. The aim of this study was to examine the feasibility of a protocol for a cluster RCT of a sit-stand desk intervention within primary school classrooms. If deemed feasible, a fully powered cluster RCT could provide valuable evidence on the effectiveness and cost-effectiveness of a sit-stand desk intervention within primary school classrooms, incorporating device-based measures of sitting and activity and a range of health and behaviour-related outcomes. The breadth and findings of the present study are essential to inform a full trial and the potential longer-term adoption of sit-stand desks in primary schools. Objectives of this pilot trial included: 1) evaluating the feasibility and acceptability of recruiting schools and children into the trial; 2) determining attrition in the trial (schools and children); 3) evaluating the acceptability of the intervention and randomisation to teachers and children; 4) determining the acceptability and completion rates of the outcome measures; 5) monitoring the occurrence of any adverse events of the intervention (or a sit-stand desk); and 6) exploring the potential of the intervention to reduce children’s device-based measurement (activPAL) of weekday sitting time (the proposed primary outcome in a full trial), and describing the proposed secondary outcome measures collected at baseline and follow-up (device-based measurement of physical activity, adiposity, blood pressure, in-class behaviour, and learning engagement).

## Methods

### Design

The detailed protocol for this pilot trial has been reported elsewhere [12]. The study was a school-based, two-armed pilot cluster RCT. Individuals (children aged 9–10 years) were the unit of analysis and schools (clusters) were stratified according to predominant pupil ethnicity (either > 50% White British pupils, or > % South Asian pupils) and randomly assigned to one of two conditions: 1) six manually adjustable sit-stand desks incorporated into the classroom environment (intervention condition), or 2) current practice (control condition). Given the intervention was delivered at the classroom level, rather than individual level, a cluster design was considered appropriate. Baseline measurements (November 2016) preceded randomisation (December 2016), and the sit-stand desks were installed into the intervention classrooms following this (February 2017, remaining until July 2017). An identical set of outcome measurements were taken from all participants approximately 7-months after baseline testing at the end of the year 5 school term (July 2017). The reporting of this trial follows the CONSORT extension statement for cluster trials [35] and the CONSORT checklist is provided as supplementary material.

### Study setting

The study was conducted in primary schools in Bradford, a northern city in England, chosen as the study location given its ethnic composition (predominantly South Asian and White British) and high levels of deprivation, health inequalities and childhood morbidity [36]. Half of all babies born in Bradford are of South Asian origin and 60% are born into the poorest 20% of the population [36]. The study setting was deemed fundamental in addressing the important issue of health inequalities, with classroom-based interventions being accessible to all children [12].

### Sample size

A recruitment target of eight primary schools, each with at least 15 child participants per class (approximately 50% of a typical class size) was set, giving a minimum total sample of 120. This exceeds the target minimum sample size recommended for pilot trials [37]. It was also assumed that this sample size should be sufficient to provide clear estimates of recruitment and follow-up to inform a full RCT [12].

### School and participant recruitment and eligibility criteria

Government-funded primary schools located in the City of Bradford were invited to participate in the study. Private and designated special educational needs schools and schools with fewer than 25 pupils in year 5 (ages 9–10 years) were not eligible. The aim was to recruit four schools with predominantly South Asian pupils (> 50%) and four with predominantly White British pupils (> 50%). Information on the ethnic composition of the schools’ pupil population was determined using local school census data [12].

The following three-stage recruitment process was adopted for schools: 1) head teachers/senior teachers were sent an email detailing the study, which included a copy of an Information Sheet for Schools; 2) 2 days after sending the email, the schools were contacted via telephone and the reception team were asked to confirm receipt of the email; 3) a follow-up telephone call was made to establish the schools’ interest or otherwise in participating in the study. A designated lead teacher was identified for each interested school who was then given full details of the study and what their involvement would entail.

Consenting schools were asked to nominate a year 5 class and were provided with invitation packs for the parents/guardians of children within these classes. All children within participating classes were eligible to take part in the evaluation. The invitation pack contained a detailed Information Sheet for Parents/Guardians, an opt-in consent form for the parent/guardian to complete and return if they were happy for their child to participate in the evaluation, and an Information Sheet for Children. Completed consent forms were returned by pupils to their teacher, who informed the research team of the children who were to be involved in the evaluation measures. At the beginning of the baseline measurement session, all methods were fully explained to children by a member of the research team at which time they were asked to provide verbal and written assent. This was requested again at the start of the follow-up measurement sessions.

### The ‘Stand Out in Class’ intervention

**Fig. 1:**
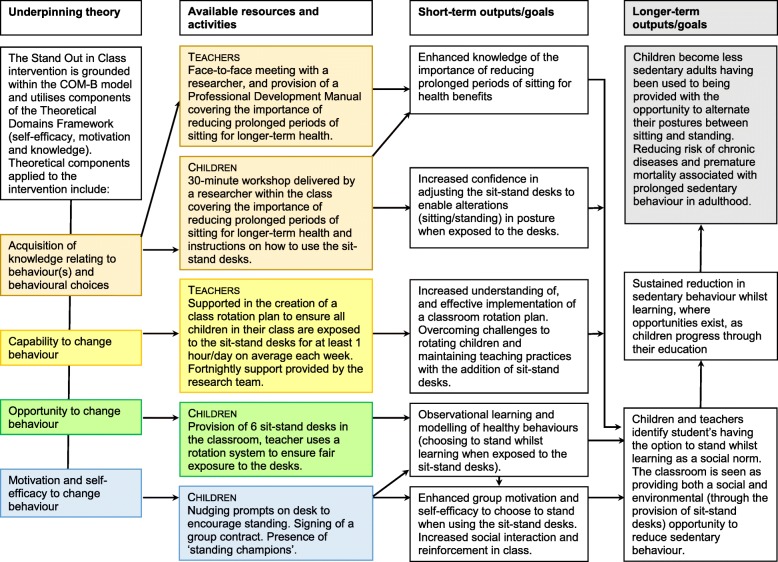
A Logic model for the Stand Out in Class intervention, applicable for a definitive trial

Six height-adjustable sit-stand desks (LearnFit, Ergotron Inc., USA) were placed in a year 5 classroom (replacing three standard desks sitting 6 children) in each intervention school for two school terms, spanning 4.5 months. The research team supported teachers in the development of a classroom rotation plan to ensure all children in their class were exposed to the sit-stand desks for at least 1 hour/day on average across the week. Stools or chairs remained in the classroom and while children were free to choose whether they sat or stood when using the sit-stand desks, they were encouraged to stand by teachers, as well as through the use of nudge prompts displayed on the desks and standing champions (i.e. one child in a class who was given the responsibility of reminding the teacher about the rotation plan) (see Fig. 1) [12].

Teachers and pupils in the intervention classrooms received training on sit-stand desk use by the research team and teachers also received a ‘Professional Development Manual’ containing information on the health benefits of reducing prolonged sitting and on correct posture when standing at the desks. The teacher manual and training focussed on encouraging adoption of the intervention, targeting key barriers and facilitators to sit-stand desk use. These were identified from: our previous work [33, 38]; the Capability, Opportunity, and Motivation to perform a Behaviour (COM-B) model within the Behaviour Change Wheel [39]; and the Theoretical Domains Framework [40] (e.g. self-efficacy, motivation and knowledge). Standardised behaviour change techniques (e.g. goal setting, instruction) [41] were also used during the training with teachers and pupils [12]. Further details of the intervention, including an overview of the intervention components and potential barriers, solutions, and hypothesised mediating processes informed by the above theoretical frameworks are reported elsewhere [12]. A logic model for the Stand Out in Class intervention, applicable for a definitive trial, is presented in Fig. 1.

### The usual practice control arm

To compare the effects of the intervention against usual practice (i.e. the provision of standard classroom desks), schools assigned to the control arm were requested to continue with their usual practice and lesson delivery; no environmental changes were made to their classrooms [12].

### Allocation to treatment groups

Schools were stratified based on the ethnic composition of their pupils. Following baseline measurements, schools within each stratum were randomised into the two study arms using an allocation ratio of 1:1, employing a randomisation list in SAS software, by an independent statistician at the Leicester Clinical Trials Unit (CTU). Two schools with predominantly South Asian pupils (> 50%) and two schools with predominantly White British pupils (> 50%) were randomised into the intervention and control arms (4 schools in each arm).

### Outcome measurements

The primary outcomes of this pilot trial were the feasibility and acceptability of the research procedures (including recruitment, data collection, randomisation, acceptability of the intervention, retention, and the presence of any adverse events) to inform the planning of a full RCT. A detailed process evaluation describing teachers’ and children’s experiences of the intervention is reported elsewhere [42]. Study uptake was monitored by recording the number of schools and pupils approached, and the number agreeing to participate (objective 1). Withdrawal rates of schools and children were recorded (objective 2). The acceptability of recruitment (objective 1), the intervention and randomisation (objective 3), and the acceptability of outcome measures (objective 4) were determined via focus groups with children and interviews with teachers. Furthermore, completion rates of the outcome measures were recorded (objective 4), along with the occurrence of any study-related adverse events (objective 5).

Interviews with teachers and focus groups with children from both trial arms were conducted approximately 1 month following randomisation to explore the acceptability of recruitment (example question: ‘*What did you think about the way that you were asked to take part in the Stand Out in Class Study?’)*, randomisation (example question: ‘*What did you think about being randomised to one of the 2 school groups in the study [control/intervention]?’*), and the measurement instruments (example question to children: *‘What was your view about wearing the thigh worn device for 7 days?*’). The acceptability of the intervention was determined through a further set of interviews (with teachers) and focus groups (with children) from the 4 intervention schools during the final month of the intervention. An example question to intervention teachers and children included: ‘*What has been your experience so far of the sit-stand desks being part of your classroom?’*

Four male (3 control group, 1 intervention) and 4 female (1 control, 3 intervention) teachers participated in the study. A total of 43 children, 22 boys and 21 girls, took part in the focus groups following randomisation (8 focus groups were conducted, 1 per school) and 24 children, 10 boys and 14 girls, participated in the focus groups towards the end of the trial (4 intervention schools only). Teachers selected children in their class for participation in the focus groups. Within the intervention classes, there may have been some overlap between children participating in the first and second focus groups (at the end of the trial). All interviews and focus groups, across both phases, used semi-structured topic guides to ensure consistency. The focus group topic guides were written in child friendly language. All interviews and focus groups were audio-recorded digitally.

Device-based sitting was measured for 7 consecutive days during each measurement period using the activPAL3 micro accelerometer (PAL Technologies, UK). This device has been shown to provide a valid measure of posture in children [43]. All activPALs were initialised and downloaded using manufacturer proprietary software (activPAL Professional v.7.2.32) and data were processed using the freely available ProcessingPAL Software (https://github.com/UOL-COLS/ProcessingPAL, version 1.1, University of Leicester, (Leicester UK)). The activPAL3 was waterproofed (using a nitrile sleeve and hypoallergenic Hypafix [BSN Medical] dressing) and participants were requested to wear the device continuously (24 h/day) on the anterior aspect of their right thigh. The device was attached using Hypafix dressing. Participants were provided with a brief diary during each monitoring period in which they were requested to document time in bed and any periods of non-wear [12]. Periods of prolonged non-wear and time in bed were removed from the data using the default algorithm rules within Processing PAL [44]. Briefly, the algorithm searches within event files (created in the activPAL Professional software) to identify prolonged bouts of behaviour (sitting, standing) within a noon-noon period. If they meet the criteria they are coded as time in bed/non-wear (no distinction). To accommodate fragmented sleep patterns, the algorithm searches around these identified bouts for other prolonged bouts of behaviour occurring after brief upright activity. If they meet the criteria, the identified bouts and the upright activity are also coded as time in bed/non-wear. Once time in bed and non-wear were excluded, a day was considered valid if it consisted of ≥8 h of waking wear data, < 95% of time spent in any one behaviour (e.g., sitting, standing, or stepping) and ≥ 500 single leg steps (i.e., ≥1000 steps) [44]. Due to the exploratory nature of this study, children were included in the analysis relating to objective 6 (exploring the potential of the intervention to reduce children’s weekday sitting time) if they had worn the activPAL for at least 8 h on at least 1 weekday at baseline and follow-up.

Proposed secondary outcomes for a future full trial included device-based measured physical activity, using the ActiGraph GT3X+ accelerometer (ActiGraph, Pensacola, FL) worn on an elasticated belt at the waist continuously (24 h/day) for 7 consecutive days, concurrently with the activPAL. The feasibility of collecting ActiGraph data, in addition to activPAL data, was examined to inform a full trial, where this device could be used as a secondary outcome to examine any positive or negative (i.e. compensatory) effects of the intervention on physical activity either during or after school hours. ActiGraphs were initialised to record data at 60 Hz. The devices were initialised and downloaded using ActiLife version 6.13.3, and the data (reintegrated into 15 s epochs) were processed using specifically developed and commercially available software (KineSoft version 3.3.20, Loughborough UK). Time spent in light (26–573 counts per 15 s epoch) and moderate-to-vigorous intensity (≥574 counts per 15 s epoch) activity were determined using the Evenson cut-points [45]. Due to the 24-h wear protocol of the ActiGraphs, a blanket removal of sleep time between 11 pm and 5.59 am was undertaken when processing these data. However, to identify periods of sleep and/or non-wear occurring outside of this time period (i.e. after 6 am and before 11 pm), the 3-axis acceleration data from the ActiGraph were used to detect periods of no movement. If these periods exceeded 20 min of zero counts, then this additional period was excluded as non-wear/sleep time. The same wear time criteria as applied to the activPAL data (a minimum of 8 h of wear on at least one weekday) was also applied to the ActiGraph data.

At each measurement point children’s height and body mass (without shoes) were measured directly using standard procedures by trained research staff. Body composition was assessed using bio-impedance analysis scales, suitable for use with children (Tanita DC-360S). Blood pressure was measured from the left arm after at least a 5 minute period of quiet sitting using a semi-automated recorder (Omron HEM-907) with a paediatric cuff, in accordance with current recommendations [46]. Three assessments were taken with each measurement separated by a two-minute rest period and the mean systolic and diastolic blood pressures recorded from the second and third assessments were calculated.

The impact of the intervention on participants’ behaviour was assessed using the Strengths and Difficulties questionnaire [47], a measure of pro-social behaviour, emotional symptoms, conduct problems, hyperactivity and peer problems, completed by teachers at baseline and follow-up. The questionnaire consists of 25 items, with five items per scale, which receive a score from 0 to 2. A total difficulties score is calculated by summing the scores from the first four scales, with higher scores indicating increased behavioural difficulties [47]. In addition, children self-reported their engagement and disaffection with their own learning via the Engagement Versus Disaffection with Learning questionnaire [48]. This questionnaire assesses behavioural engagement and behavioural disaffection, using five items each, along with emotional engagement, using five items, and emotional disaffection, using 12 items. Each item is scored on a 1 to 4 scale, with higher values indicating increased levels of engagement and reduced disaffection. Mean scores are calculated across the two engagement and disaffection categories to provide an overall indication of engagement and disaffection levels [49].

Children furthermore completed the Paediatric Quality of Life Inventory (PEDS-QL) [50] and EuroQol 5-dimension Youth (EQ-5D-Y) [51] at each measurement point to provide a measure of self-reported quality of life to inform an economic analysis in a full trial. Basic demographic information (sex, age, ethnicity) were reported by children at baseline. Full details of all measurement instruments, along with information on their validity has been reported elsewhere [12].

### Quantitative and qualitative analyses

#### Trial feasibility and acceptability

As this was a pilot trial, the primary analyses (the purpose of which was to assess the feasibility of conducting a cluster RCT of a sit-stand desk intervention within primary school classrooms) mainly utilised descriptive statistics summarising: the number of schools approached, the number agreeing to participate, and the proportion of children within each school with parental/guardian consent, and giving their assent, to participate in the study evaluation (objective 1); retention rates (schools and children) (objective 2); outcome measure completion rates and compliance (objective 4); and the documentation of any study-related adverse events (objective 5).

The acceptability of recruitment (objective 1), randomisation and the intervention (objective 3), along with the acceptability of the outcome measures (objective 4) were determined through qualitative analyses of the pupil focus groups and teacher interview data. Audio recordings were transcribed verbatim with anonymisation of all personal data. To address the objectives within the present paper, sample quotes which reflect common responses across the questions asked are provided (a detailed process evaluation is reported elsewhere [42]). Extracts from the focus groups and interviews are labelled to indicate the participant (Child/Teacher), group (I = intervention, C = control) and school (number 1–4 within each trial arm).

#### The potential of the intervention to reduce children’s weekday sitting time, and a summary of the proposed secondary outcomes for inclusion in a full trial (objective 6)

An objective of this study was to examine the potential of the intervention to reduce children’s weekday sitting time (the proposed primary outcome in a full trial). As the number of clusters was low, cluster summary statistics were used rather than multi-level modelling [52, 53]. A weighted linear regression model compared the change in mean weekday sitting time between follow-up and baseline between control and intervention arm participants. The model was adjusted for baseline total daily sitting time on school days and average weekday wear time across the two measurement points. Subsequent models adjusted for the season in which the baseline and follow-up measures were taken. Since the variables in the regression model reflect cluster means rather than individual observations, an analytically weighted least squares method of estimation was used, where cluster sizes were the weights. The results from this analysis should, however, be treated as preliminary and interpreted with caution given the lack of statistical power [54, 55]. Statistical analyses were undertaken using Stata version 15.1 (StataCorp, Texas, USA), and were validated by an independent trial statistician at the Leicester CTU.

Descriptive statistics were calculated to summarise the proposed secondary outcomes (device-based measured time spent in light intensity and moderate-to-vigorous intensity activity on weekdays, adiposity, blood pressure, behaviour, and learning engagement) measured at baseline and follow-up.

## Results

### Trial feasibility and acceptability

Twenty-four eligible schools were approached and of these the target number of eight schools consented to participate, with the overall recruitment rate being 33% (95% CI: 16 to 55%). Twelve schools did not consent to join the study (50%) and four did not respond to the initial email (17%). All eight participating schools completed the trial (100% retention). Data from the 2016–2017 school census [56] show that the proportion of children eligible for free school meals was similar across the recruited schools and the declined schools (mean: 17.1% [range: 2.3, 26.4%] vs. 17.4% [9.6, 28.5%]), with these values being higher than the national average of 14.8% in 2016–2017.

**Fig. 2:**
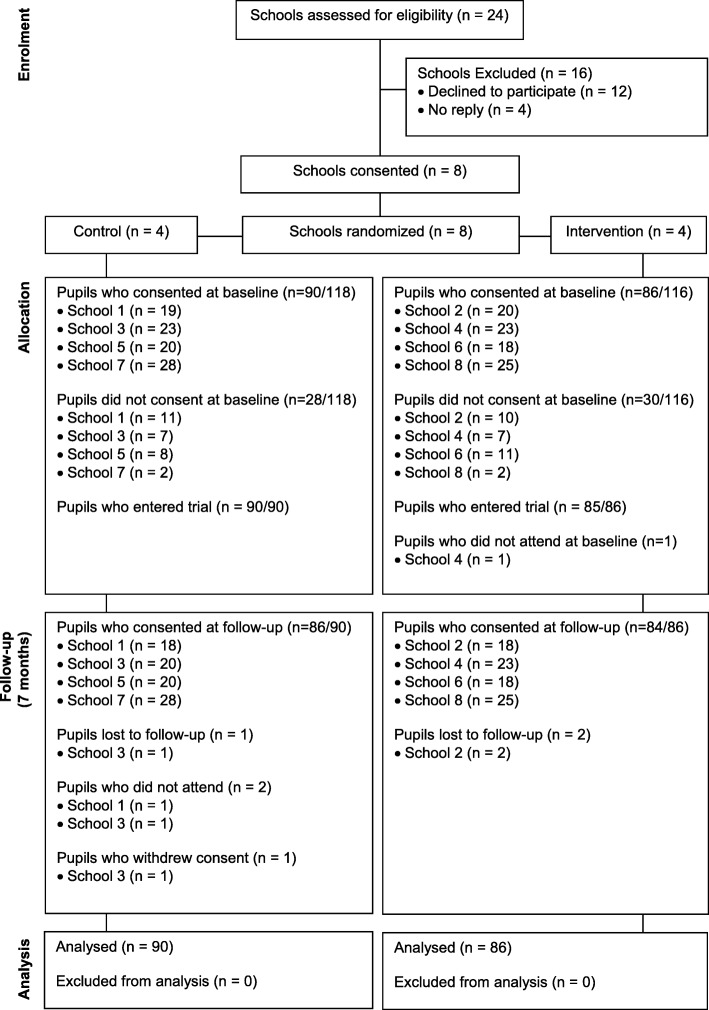
A CONSORT Diagram for the Stand Out in Class pilot cluster RCT

The proportion of pupils at the eight schools with parental consent to participate in the trial evaluation was 75% (176 out of 234), exceeding the target minimum sample of 120 [12]. At follow-up, retention of participating children was 97% (170 out of 176). A CONSORT flow diagram is shown in Fig. 2. Two pupils in the control group were unable to provide follow-up measures as they were absent from school on the days they were taken. Three children (1 control, 2 intervention) moved away from the area during the study and hence changed schools. One control group participant withdrew their assent prior to the follow-up measures. The demographic characteristics of the participating children at baseline are shown in Table 1.

**Table 1:** Demographic characteristics of the participating children, by group and total sample

		Control (*n* = 90)	Intervention (*n* = 86)	Overall (*n* = 176)
Sex, n (%)	Male	50 (55.6%)	48 (55.8%)	98 (55.7%)
	Female	44 (44.4%)	38 (44.2%)	78 (44.3%)
Ethnicity, n (%)	White British	18 (20.0%)	45 (52.3%)	63 (35.8%)
	South Asian	59 (65.6%)	26 (30.2%)	85 (48.3%)
	Other	13 (14.4%)	15 (17.4%)	28 (15.9%)
Age	Mean (SD)	9.3 (0.5)	9.3 (0.4)	9.3 (0.5)

Completion rates of the proposed outcome measures for inclusion in a full RCT at baseline and follow-up are shown in Table 2. The table also displays the proportion of children providing valid activPAL and ActiGraph data on at least 1, 2, 3, 4 and all 5 weekdays.

**Table 2:** Total sample outcome measure compliance and completion rates at baseline and follow-up

	Baseline	Follow-up	Both baseline and follow-up
activPAL data on weekdays^a^			
≥ 1 valid day	80.1%	76.1%	63.1%
≥ 2 valid days	74.4%	66.5%	51.7%
≥ 3 valid days	65.3%	53.4%	39.2%
≥ 4 valid days	54.5%	42.6%	27.3%
5 valid days	18.2%	16.5%	5.7%
ActiGraph data on weekdays^a^			
≥ 1 valid day	94.3%	87.5%	83.5%
≥ 2 valid days	89.8%	78.4%	73.3%
≥ 3 valid days	85.2%	65.3%	58.0%
≥ 4 valid days	75.0%	50.0%	42.6%
5 valid days	25.6%	11.4%	5.1%
Anthropometric measures	98.9%	95.5%	94.3%
Body composition	98.9%	94.9%	93.8%
Blood pressure	77.8%	89.8%	70.5%
Engagement vs Disaffection with Learning (child reported)	97.7%	96.0%	93.8%
Strength and Difficulties questionnaire (teacher reported)	91.5%	94.9%	90.3%
PEDS-QL	83.0%	93.2%	83.0%
EQ-5D-Y	94.6%	94.6%	94.6%

^a^A valid day for the activPAL and ActiGraph constituted at least 8 h of wear on a weekday

No serious adverse events were reported throughout the duration of the trial. Specifically, there were no adverse effects associated with the intervention that related to musculoskeletal discomfort and/or disruption to the classroom or to reported learning.

All eight teachers expressed high satisfaction with the recruitment protocol, with all stating the study had been clearly explained:

*“Yeah, it was very well explained and the ideas and the concept behind what you were doing, so I had no hesitation accepting really.”* (Teacher, C1)

Teachers also commented that the recruitment approach was appropriate and suitable for children:

*“It worked well. I think you got quite a good uptake … as a class, so obviously what you were sending out and the conversations you were having with the children got them quite enthused. I think with them, with the children they’re doing something scientific because they all sort of really love science, the idea of doing something scientific with scientists is like “yay!” So they jumped on that.”* (Teacher, C2).

Children across all focus groups reported that recruitment had been positive for them, the study made clear, and that everyone had a choice to participate:

*“It was good because once I got the letter, I didn’t understand what it was. [Researchers] told us about the letter and our teachers told us about it and told us to tell our mum if we want to go or not because you first need permission off your mum, and that’s why it was a good process because you got told three times.”* (Child, I1).

*“It’s more like you get to choose to take part and if you don’t want to then it doesn’t matter.”* (Child, I3).

When asked about the acceptability of randomisation, all teachers and children expressed a clear understanding of why randomisation had occurred. Whilst control group teachers and children were disappointed not to have worked with the sit-stand desks, they considered their participation in the trial to be positive and important:

“*Well, I completely understand why you need to have a control. You know, we teach the children, that certain investigations need a control, you need something to compare it against* … ” (Teacher, C3).

*“Because then you can look at the schools that have the tables and the schools that didn’t and look at the difference on health.”* (Child, I3).

With regards to the acceptability of the activPAL (as a primary outcome measure for a full trial), the most common theme identified from the responses related to issues with the medical dressing used (Hypafix® transparent) to attach the monitor. This reportedly caused a minority of children to suffer from itchiness, soreness and discomfort, and led to some class disruption:

*“Yeah, it was a bit faffy. Some of the children did complain about getting a bit of a rash, but they like to complain anyway, so it was a bit… I don’t want to use the word chaotic, but that was more to do with the fact that the kids were constantly interested by them so they were focused on them … ”* (Teacher, I1)

However, other teachers did not perceive the medical dressing to be particularly problematic as only a few children had been affected:

*“ … there were only a few complaints [about the dressing] … ”,* (Teacher, C2)

“*Only a couple of them had a little reaction to it.”* (Teacher, I4*)*

During the focus groups with children, 10 out of 43 reported feeling some discomfort related to the activPAL:

*“When you tried to take it off it really hurt.”* (Child, C3)

*“When I took it off I had a bit of like a little rash or a few spots, from the underneath because my leg got quite sweaty.”* (Child, C1).

In contrast to the activPAL, the ActiGraph was regarded as a more acceptable device for children to wear by all teachers and most children (38/43):

“*It didn’t really annoy me at all and it felt like nothing was even there.*” (Child, C4)

The focus groups and interviews with intervention children and teachers conducted towards the end of the study period revealed that the intervention was generally well accepted by children and teachers. All teachers expressed that the desks had become part of their classroom, and that any initial concerns they had had regarding the desks causing a distraction had not materialised:

*“Yeah, well for me I'm now used to them so before, I think for the first month or so, I was kind of looking at them as to how would they work, how well would they work with the children, would it just be a distraction for them, but now it's, it's kind of just the norm for the children, and we're kind of, we're used to them and every week when we rotate round we, we just do it steadily.”* (Teacher, I2).

The children felt having the desks in their classroom had been very positive, with key themes including changing behaviour for the better, liking having the option to stand, and appreciating the increased personal working space afforded by the desks:

*“they really change boys’ behaviours because some boys, not me, are fidgety so it’s good for them to stand up*.” (Child I4)

*“I like it because, like, every time you don’t feel comfortable while sitting down, you could just stand up and then you might feel more comfortable.”* (Child, I3).

*“It’s like it’s a lot better than our tables because when we do our work, sometimes Miss says, sit down to do our work but then now with the stand-up-sit-down tables we can stand up more because I like working when I stand up especially when it’s stuff like art and stuff like that where you have to draw.”* (Child, I4).

*“I liked it because it was only for one person to sit on, for each table. Because normally, when we have to share a table, there’s not enough space.”* (Child, I2).

### The potential of the intervention to reduce children’s weekday sitting time

An objective of this pilot trial was to examine preliminary evidence of the effectiveness of the intervention in changing mean weekday sitting time, as the intervention was school based. Total school day/weekday sitting time was chosen as this encompasses school hours and out of school hours, and factors in any potential compensatory effects of the intervention (i.e. increases in sitting after school). Table 3 displays the descriptive statistics for all activPAL variables recorded throughout waking hours on weekdays for the control and intervention groups.

**Table 3:** Descriptive statistics for the activPAL variables measured throughout waking hours on weekdays

Waking hours on weekdays	Baseline		Follow-up		Change	
	Control (n = 57)	Intervention (*n* = 52)	Control (*n* = 57)	Intervention (*n* = 52)	Control (n = 57)	Intervention (n = 52)
Wear time (min/day)	836.3	843.8	830.9	835.4	−3.7	−8.4
	(88.5)	(47.8)	(78.6)	(64.2)	(121.6)	(62.3)
Time spent sitting (mins/day)	520.1	514	504.4	472.0	−15.2	−42.0
	(83.6)	(61.5)	(94.0)	(73.5)	(107.5)	(76.6)
Time spent standing (mins/day)	179.9	195.4	176.5	197.1	−3.0	1.6
	(58.6)	(38.7)	(45.7)	(49.4)	(50.2)	(52.0)
Time spent stepping (min/day)	136.3	134.4	150.0	166.4	14.4	32.0
	(44.9)	(30.4)	(42.1)	(41.9)	(44.8)	(41.1)
Percentage of wear time spent sitting (%)	62.4	60.9	60.5	56.5	−2.0	−4.3
	(8.8)	(5.9)	(8.6)	(8.2)	(8.7)	(8.6)
Percentage of wear time spent standing (%)	21.4	23.2	21.5	23.6	0.1	0.4
	(6.3)	(4.5)	(6.1)	(5.7)	(5.9)	(5.8)
Percentage of wear time spent stepping (%)	16.2	15.9	18.1	19.9	1.9	3.9
	(4.7)	(3.5)	(4.8)	(4.6)	(4.6)	(4.6)
Number of sit to stand transitions	102.5	106.4	104.1	106.2	1.6	0.2
	(28.7)	(23.6)	(26.5)	(21.4)	(25.0)	(20.5)
Number of days worn	3.7	3.5	3.2	3.5	−0.5	0.0
	(1.3)	(0.9)	(1.2)	(1.4)	(1.4)	(1.8)

Data are presented as the mean (SD). This table includes data from participants who wore the activPAL device with a minimum valid wear time of 8 h each day on at least one weekday at baseline and at 7-months follow-up.

The weighted linear regression model applied revealed the mean difference in change in sitting time was −30.6 min/day (95% CI: −56.42 to −4.83) for the intervention group, relative to the control group. The addition of baseline season of activPAL data collection to the weighted linear regression model did not affect the difference in sitting time between groups. When follow-up season was included in the model the adjusted difference in sitting time between groups was − 26.64 min/day (95% CI: − 73.08 to 19.79).

Table 4 displays the descriptive statistics for all ActiGraph variables recorded throughout waking hours on weekdays for the control and intervention groups. Both groups demonstrated small changes in light intensity physical activity and moderate-to-vigorous intensity physical activity (MVPA) over the follow-up period. Descriptive statistics for the anthropometric, blood pressure and questionnaire measures (Engagement and Disaffection with Learning and the Strengths and Difficulties questionnaire) collected from participants at baseline and follow-up are shown in Table 5. The changes seen in the anthropometric measurements over the follow-up period are reflective of typical growth-related changes in children of this age. There were no noticeable between-group differences in the mean changes in learning engagement and disaffection scores over the trial period, and a small decrease in the total difficulties score (indicating improved behaviour) in the intervention group relative to the control group over the follow-up period.

**Table 4:** Descriptive statistics for the ActiGraph variables measured throughout waking hours on weekdays

Waking hours on weekdays	Baseline		Follow-up		Change	
	Control (*n* = 74)	Intervention (*n* = 72)	Control (*n* = 74)	Intervention (*n* = 72)	Control (n = 74)	Intervention (n = 72)
Wear time (min/day)	885.1	882.6	827.7	852.9	−57.4	−29.7
	(90.5)	(84.5)	(134.1)	(106.8)	(125.9)	(118.0)
Time spent in light PA (mins/day)	378.2	383.5	364.3	392.7	−13.9	9.3
	(61.9)	(68.6)	(81.2)	(70.8)	(74.4)	(78.3)
Time spent in MVPA (min/day)	40.0	37.4	40.7	45.7	0.7	8.3
	(20.5)	(17.9)	(30.9)	(24.7)	(24.5)	(20.0)
Percentage of wear time spent in light PA (%)	43	43.4	44.0	46.0	1.1	2.6
	(6.4)	(6.2)	(6.9)	(6.0)	(5.5)	(5.6)
Percentage of wear time spent in MVPA (%)	4.6	4.3	5.0	5.4	0.5	1.1
	(2.3)	(2.1)	(3.8)	(2.7)	(2.8)	(2.2)
Number of days worn	3.8	3.6	2.8	3.2	−1.0	−0.4
	(1.4)	(1.3)	(1.5)	(1.6)	(1.3)	(1.3)

Data are presented as the mean (SD). This table includes data from participants who wore the ActiGraph device with a minimum valid wear time of 8 h each day on at least one weekday at baseline and at 7 months follow-up.

**Table 5:** Anthropometric, blood pressure and questionnaire measurements

	Baseline		Follow-up		Change	
	Control (n = 90)	Intervention (*n* = 84)	Control (n = 85)	Intervention (n = 83)	Control (*n* = 85)	Intervention (*n* = 81)
Height (cm)	140.5	138.3	144.0	141.3	3.3	2.9
	(6.6)	(6.2)	(6.8)	(6.4)	(1.7)	(1.0)
Body mass (kg)	36.3	35.0	39.2	37.7	3.0	2.7
	(9.5)	(7.8)	(10.6)	(8.7)	(1.7)	(1.7)
Percent body fat – Girls^a^	24.4	23.6	23.7	25.0	−0.7	0.5
	(8.4)	(8.1)	(9.1)	(8.3)	(2.1)	(2.8)
Percent body fat - Boys^a^	20.6	19.9	20.7	19.0	0.4	−0.9
	(8.9)	(6.9)	(8.9)	(6.6)	(2.6)	(2.4)
BMI (kg/m^2^)	18.2	18.2	18.7	18.8	0.6	0.6
	(4.0)	(3.3)	(4.1)	(3.5)	(0.8)	(0.7)
Systolic blood pressure (mmHg)^b^	102.5	102.8	107.3	110.5	5.1	10.2
	(11.8)	(15.2)	(11.7)	(11.2)	(15.8)	(17.8)
Diastolic blood pressure (mmHg)^b^	66.1	67.3	66.3	68.4	0.2	2.4
	(10.2)	(14.1)	(9.5)	(9.7)	(12.1)	(16.2)
Engagement and Disaffection with Learning questionnaire sub-scale scores (child reported)						
	Control (n = 90)	Intervention (*n* = 82)	Control (n = 86)	Intervention (n = 83)	Control (n = 86)	Intervention (*n* = 80)
Overall Engagement	3.4 (0.5)	3.4 (0.5)	3.3 (0.6)	3.3 (0.5)	−0.1 (0.6)	−0.1 (0.5)
Overall Disaffection	3.1 (0.7)	3.1 (0.7)	3.2 (0.7)	3.1 (0.6)	0.1 (0.7)	0.0 (0.6)
Strengths and Difficulties questionnaire (teacher reported)						
	Control (n = 83)	Intervention (n = 78)	Control (*n* = 83)	Intervention (n = 84)	Control (n = 81)	Intervention (*n* = 78)
Total difficulties score	6.2 (5.7)	9.2 (7.6)	6.9 (6.0)	7.8 (6.6)	0.6 (4.6)	−1.3 (4.5)

Data are reported as the mean (SD). ^a^Percent body fat sample sizes: girls, control *n* = 40, intervention *n* = 35; boys, control *n* = 50; intervention *n* = 49. ^b^The sample size for the change in blood pressure measurements reduced to 54 control participants and 49 intervention participants.

## Discussion

The purpose of this study was to undertake a pilot cluster RCT to test the feasibility and acceptability of conducting and evaluating a school-based sit-stand desk intervention. The findings confirmed that recruitment and attrition rates were acceptable to support progression to a full trial, most outcome measures were acceptable, and the intervention was well received. However, improvements to compliance with protocols for assessing the proposed primary outcome (activPAL-determined sitting time) are needed. Furthermore, preliminary evidence demonstrated the potential of the intervention in reducing children’s weekday sitting time, although the changes observed were not as large as those seen previously within the same setting within a 9-week non-randomised controlled study conducted in just one school [33].

The uptake into the study by schools (33% of those approached) is similar to recruitment rates seen in other primary school-based interventions located in the same region [57] and elsewhere in England [58]. Whilst all eight recruited schools were located predominantly in urban areas within the Bradford metropolitan district, the study was effective in recruiting a diverse range of schools in terms of the ethnic composition of their pupils within a relatively deprived setting. Within the participating schools, parental consent and pupil assent to participate was obtained for 75% (*n* = 176) of eligible pupils, exceeding our target minimum sample size (120 participants) [12]. Furthermore, school and participant retention rates within the trial were high (100 and 97% respectively). Overall, these findings demonstrate the feasibility of recruiting and retaining schools and participants into a school-based sit-stand desk RCT and suggest good interest and recognition of the importance of the study by participating schools. Whilst schools have been identified as important environments for health promoting interventions [22], the challenges of recruiting schools and children, particularly via opt-in consent procedures (as adopted herein), and in retaining participants, have been highlighted [59].

Most outcome measures were regarded as acceptable by children and teachers. Of the physiological measures, lower compliance rates were seen for blood pressure, with some children stating during the assessments that they found this measure uncomfortable. Whilst modest (63%), the proportion of children providing valid activPAL data in the present study is higher than that observed previously in the same study setting [33], and similar to that in a recent sit-stand desk RCT in Belgian children [26]. The main issue faced was with the medical dressing (Hypafix [BSN Medical]) used to attach the activPAL, with this reportedly causing irritation on the leg for some children. In the present study we adopted a 24-h wear protocol with the anticipation that the hypoallergenic dressing would stay on the skin for a number of days, and not require children to frequently remove the device (and dressing), with the purpose of reducing participant burden. However, this did not prove to be very effective as a number of children requested additional medical dressing throughout the monitoring periods to enable them to re-attach the activPAL after removal. Other researchers have enclosed the activPAL in a small pocket in an adjustable elasticised belt worn at the mid-anterior position of the thigh throughout waking hours only, removing it for water-based activities. This approach has been used successfully (85% compliance) in cross-sectional research [60] and is worth exploring ahead of a full trial. Evidently, further research is needed on the attachment options for the activPAL in children to improve compliance. In comparison to the activPAL, compliance rates for the waist-worn ActiGraph were higher (83%) and this device was reasonably well accepted by children.

The intervention was well received by teachers and children, and towards the end of the intervention teachers commented on how the desks were regarded as part of the norm within their classrooms. This positive finding suggests teachers are both prepared and capable of adapting their teaching style and willing to make modifications to their classroom environments. Some children reported that they felt the desks improved behaviour within the classroom. These findings are consistent with others who have concluded that sit-stand desks can be introduced into the classroom environment without having a negative impact on student learning, behaviour, musculoskeletal comfort, or causing classroom disruption [28, 29, 31, 61, 62]. The absence of any negative impacts of sit-stand desks on these outcomes are likely to be of particular interest to schools considering adopting these desks in the future. Further, the potential positive effects observed within this study on pupil behaviour and increases in pupil autonomy (having the choice of sitting or standing) are even more encouraging and support further testing of this intervention.

Preliminary analyses demonstrated the potential of the intervention in reducing children’s weekday sitting time, with the intervention group reducing their total weekday sitting time by more than 30 min/day relative to the control group. No data currently exist in children to inform the magnitude of a reduction in sitting time needed to bring about changes in health markers. This information will be vital in the future to inform public health messaging. Data from adults however have indicated that reallocating just 30 min of sedentary time per day to light movement is associated with a 2–4% improvement in cardiometabolic biomarkers [63]. An earlier meta-analysis of RCTs and non-RCTs delivered in the school or home environment reported an overall decrease in children’s sedentary behaviour of 18 mins/day [64]. The preliminary findings from this study hold promise, therefore, and support the need for further RCTs examining the impact of sit-stand desks in the classroom environment. The reduction in sitting time observed in the current pilot RCT is also greater than that reported in a recent sit-stand desk RCT conducted in primary school children in Belgium where, relative to the control group, the intervention group experienced a reduction in daily sitting of 13.5 min/day over the 8–12 week intervention period [26]. In the Belgian study however, only three sit-stand desks were placed in the intervention classrooms and pupils were exposed to these desks for an average of 60 min/week, which likely explains the differences in findings.

When a bank of sit-stand desks are included within the classroom environment, as in the present study, the Belgian study [26] and in our earlier study [33], the creation and successful implementation of a regular rotation plan is important in order to maximise pupil exposure to the sit-stand desks. In our previous small study, the teacher was very effective in rotating pupils daily around the classroom to ensure equal exposure to the desks of approximately one hour/day on average, and this led to a large reduction in mean weekday sitting time (81 mins/day). In the present study, our intervention instructed teachers to rotate children daily, however some intervention teachers trialled different rotation options which may have reduced the overall exposure to the desks and the subsequent impact of the intervention and explain the differences between our study findings. This has been explored further as part of the process evaluation (reported elsewhere) [42]. It was observed in the present study that daily sitting time appeared to be replaced predominately with stepping time, as opposed to standing time, in the intervention group at follow-up. This finding contrasts to that seen in adult samples within RCTs implementing sit-stand desks in the workplace, where sitting time is predominately replaced with standing time [65, 66]. A possible explanation for this finding could be that children may be less likely to stand still when using a sit-stand desk, and hence some stepping movement could be recorded by the activPAL. Furthermore, rotating children around the class to facilitate their exposure to the sit-stand desks may also increase overall movement levels.

### Study limitations and strengths

Delays experienced at the start of the study meant that the duration of the intervention was shorter than originally proposed (2 school terms as opposed to 3 terms). Nevertheless, the overall duration was deemed appropriate to provide evidence of the feasibility and acceptability of the study protocol to inform the planning of a full trial. A further limitation was the relatively poor compliance to the activPAL protocol. Despite schools being stratified by their pupils ethnicity (either > 50% South Asian pupils, or > 50% White British pupils) with two schools from each stratum being randomised into the intervention and control arms, the balance between South Asian and White British participants across the two arms was not equal. This discrepancy was likely due to the ethnic composition of children in the individual classes involved in the trial, and discrepancies in consent from the individuals rather than an overall imbalance across the schools.

A key strength of this study includes the multi-method approach which enabled a thorough evaluation of all trial procedures. Other strengths are that the intervention was based on a theoretical framework, and its development was informed by the literature [23–25], our early work [33, 38], and public involvement (including focus groups with children and interviews with teachers and head teachers during the planning stages, along with ongoing consultation with teachers throughout the trial). The study setting, in terms of its location, associated demographics and school context, was a further strength of the trial. As noted earlier, Bradford was purposely chosen as the study location given its ethnic composition (predominantly South Asian and White British) and high levels of deprivation, health inequalities and childhood morbidity [36]. The characteristics of the participating schools suggest they were largely representative of schools within the Bradford metropolitan district which enabled us to pilot this intervention under challenging circumstances. The acceptability and feasibility findings of this study therefore suggest that this trial would likely be feasible within other schools. The accessibility of the classroom-based setting to all children is furthermore important for addressing health inequalities. Forty-eight percent of the present sample were of South Asian ethnic origin. With the emergence of an increased cardiometabolic health risk profile observed in British South Asian children, in comparison to white British children [15], early health promotion interventions like this in such higher-risk groups, could be an important strategy for reducing ethnicity-related health inequalities later in life.

## Conclusions and recommendations

The present study demonstrated that recruitment and retention rates were adequate, and randomisation, the measurement procedures and intervention were generally acceptable to participants. Some modifications to the protocol are needed to ensure the successful conduct of a future RCT, particularly around improvements to the activPAL wear protocol. Preliminary evidence from this study has demonstrated the potential of the intervention to reduce children’s weekday sitting time but more work is needed with teachers to create an acceptable classroom rotation plan to ensure pupil exposure to the sit-stand desks is maximised. The findings from this pilot cluster RCT therefore support the conduct of a full trial designed to evaluate the effectiveness and cost-effectiveness of a sit-stand desk intervention within the primary school setting on children’s sedentary behaviour, markers of health and behavioural outcomes. As suggested elsewhere [26, 31], a full trial should be conducted over a minimum of one academic year. Such a trial could provide novel and robust evidence of the longer-term health and education impacts of this intervention.

## Supplementary information

**Additional file 1.** CONSORT 2010 checklist of information to include when reporting a pilot or feasibility trial.

**Additional file 2.** The TIDieR (Template for Intervention Description and Replication) Checklist.

## Data Availability

Data supporting the results reported in this paper are stored at Loughborough University and are available upon request by contacting the first author.

## Abbreviations

RCT: Randomised controlled trial
CI: Confidence interval
METs: Metabolic equivalents
UK: United Kingdom
CONSORT: Consolidated Standards of Reporting Trials
USA: United States of America
COM-B: Capability, Opportunity, and Motivation to perform a Behaviour
CTU: Clinical Trials Unit
Hz: Hertz
PEDS-QL: Paediatric Quality of Life Inventory
EQ-5D-Y: EuroQol 5-dimension Youth
SD: Standard deviation
MVPA: Moderate-to-vigorous physical activity
cm: Centimetres
kg: Kilograms
BMI: Body mass index
